# Distinguishing Homokaryons and Heterokaryons in Medicinal Polypore Mushroom *Wolfiporia cocos* (Agaricomycetes) Based on Cultural and Genetic Characteristics

**DOI:** 10.3389/fmicb.2020.596715

**Published:** 2021-01-25

**Authors:** Shoujian Li, Qi Wang, Caihong Dong

**Affiliations:** ^1^State Key Laboratory of Mycology, Institute of Microbiology, Chinese Academy of Sciences, Beijing, China; ^2^College of Life Sciences, University of Chinese Academy of Sciences, Beijing, China; ^3^Hubei Provincial Hospital of Traditional Chinese Medicine, Wuhan, China

**Keywords:** *Wolfiporia cocos*, culture characteristics, homokaryon, heterokaryon, fruiting body

## Abstract

The sclerotia of *Wolfiporia cocos* are a kind of traditional medicine and food with excellent benefits and are widely used in China, Japan, and other Asian countries. The mating system of fungi is not only of practical importance for breeding but also has profound effects on genetic variability and molecular evolution. However, the lack of clamp connections in *W. cocos* increases the difficulty of research on mating systems. In this study, homokaryons and heterokaryons were distinguished by comparing the characteristics of culture, fruiting tests, and molecular markers, which was further demonstrated by k-mer analysis based on Illumina sequencing. Uninucleate, binucleate, and nuclei-free condition basidiospores of *W. cocos* were observed, and binucleate basidiospores were the most predominant. Brown-type colonies, slow growth rates in both PDA medium and sawdust substrate, and neutral pH after the growth of mycelia and unfruiting were found to be the morphological and growth characteristics of homokaryotic strains. Primers SSR37 and 38 were screened to identify homokaryons. K-mer analysis based on Illumina sequencing exhibited different heterozygous ratios for homokaryons and heterokaryons. The results revealed that pseudo-homothallism was the predominant mode of reproduction in the Chinese population of *W. cocos*, and heterothallism also existed in all probability. This study will be helpful for the cross-breeding of this precious medicinal mushroom and for understanding its evolution and population structure.

## Introduction

*Wolfiporia cocos* (F.A. Wolf) Ryvarden & Gilb., also called tuckahoe, grows on the roots of pines and is distributed in East Asia, India, and some American states (Weber, 1929). Dried sclerotia are widely used as traditional crude drugs in China, South Korea, and Japan, whereas they are used as food by Native Americans. It was first recorded in *Shennong Bencao Jing*, which is the earliest authoritative monograph on pharmacy in China (Gu, 2007), and also in each edition of the *Chinese pharmacopoeia*. Sclerotia have been demonstrated to have multiple activities, including immunomodulatory (Pu et al., 2019; Tian et al., 2019), antitumor (Shi et al., 2017; Liu et al., 2019), antioxidant (Wang et al., 2016), and anti-inflammatory activities (Chen et al., 2020), as well as regulation of intestinal flora (Feng et al., 2019).

*Wolfiporia cocos* is a wood-decay fungus with a subterranean growth habit. The sclerotia of *W. cocos*, tuber-like structures (Supplementary Figure S1), occasionally occur on the roots of diverse species of *Pinus* in the field and can be artificially cultivated with pine logs (Wang et al., 2002; Kubo et al., 2006; Jo et al., 2017). During the life cycle, fruiting bodies were rarely found in the field; therefore, the scientific description of the sclerotia was given in 1822 (von Schweinitz, 1822), but the sexual stage remained unknown until 1922 (Wolf, 1922). Fruiting bodies can form on both cultivated sclerotia and mycelia (Xu et al., 2014). There is no evidence of rhizomorphs in the soil surrounding the sclerotia (Wolf, 1922), and the life cycle of this fungus is yet to be clarified (Li et al., 2019).

The sclerotia of *W. cocos*, known as “*Fuling*,” have been commercially cultivated in China for 500 years (Wang et al., 2002) as well as in Japan, South Korea (Kubo et al., 2006; Jo et al., 2017), and Africa. It was estimated that the annual yield of the sclerotia of *W. cocos* was about 35,000 tons in China, and the export volume was about 4,200 tons, in 2019, and it had an export value of more than 20 million USD^1^.

Similar to the cultivation of some mushrooms, sclerotium cultivation of *W. cocos* includes spawn production, preparation of pine logs, inoculation, management, and sclerotium harvesting (Supplementary Figure S1). With the development of the scale of sclerotium cultivation, long-term asexual reproduction leads to a universal degeneration of cultivated strains, which results in a decrease in the sclerotium yield or no sclerotium production (Xu et al., 2014). Breeding of superior strains has become increasingly important for this fungus. Understanding the mating system and mode of reproduction of fungi are prerequisites for breeding.

Generally, three mating systems are known in fungi: homothallism (self-compatible), bipolar heterothallism, and tetrapolar heterothallism (Kües et al., 2011). The lack of clamp connections in *W. cocos* (Igari et al., 1995), and the fact that pairing between single spore isolates (SSIs) in culture gave little indication of the formation of secondary or heterokaryotic mycelia, increased the difficulty of the mating system research. *W. cocos* was reported to be of bipolar heterothallism (Shan and Wang, 1987) or tetrapolar heterothallism (Yan, 1992) in the early reports. Some SSIs can fruit; therefore, secondary homothallism has been proposed for *W. cocos* (Li et al., 2002; Xiong et al., 2006). Meng (2012) reported it as bipolar heterothallism based on the fruiting of SSIs and the pairing test. Using a population genetic approach, James et al. (2013) demonstrated that the species was likely to be bipolar because the polymorphism at the homeodomain-encoding genes (HD) locus was much greater than that observed at the pheromones and G protein-coupled pheromone receptor gene (P/R) locus. Further phylogenetic analysis indicated that *W. cocos* was within a clade containing only bipolar species (James et al., 2013). The different results of the mating system of *W. cocos* may be owing to the negligence of homokaryons and heterokaryons in SSIs since the binucleate basidiospores were observed with a ratio of 75.3% (Li et al., 2002). The genome of the *W. cocos* strain MD-104 has been published based on the SSI SS10 (Floudas et al., 2012). However, the SSI germinated by binucleate spores may result in heterokaryotic strain, which may affect genome assembly.

The discrimination between homokaryons and heterokaryons is the key to deciding the mating system and acquiring high-quality genome information in *W. cocos*; however, it is difficult and time-consuming. In some mushrooms, it was found that homokaryotic and heterokaryotic isolates could be reliably distinguished using the combined characteristics including the culture morphology, isozyme, and molecular markers, but not any single character (Hansen, 1979). In *Agaricus bisporus*, the traditional identification of homokaryotic strains is generally dependent on their morphological characteristics and isozyme differences (Li and Fang, 2002). The SSIs identified as homokaryons exhibited an inability to produce fruiting bodies and a slower and commonly less vigorous spawn run process, and they grew more slowly than heterokaryons in *A. bisporus* (Nazrul et al., 2010). Homokaryons grew more slowly, appearing more uniform in culture than heterokaryons, and could not form basidiocarps in culture in *Phellinus weirii* (Hansen, 1979).

Some molecular screening methods have been used to distinguish homokaryons and heterokaryons in *A. bisporus*, including inter-simple sequence repeats (ISSR; Nazrul and Bian, 2011; Sharma et al., 2014) and random amplified polymorphic DNA (RAPD; Kavousi et al., 2008). Mating-type genes have been used as molecular markers to identify homokaryons and heterokaryons in *Volvariella volvacea* (Xiong et al., 2014). Multilocus genotype tests revealed that *Agaricus subrufescens* was amphithallic with percentages of heterokaryotic single spore progenies of 75% for the Thai strain and around 40% for the Brazilian and French strains (Thongklang et al., 2014). Two cleaved amplified polymorphic sequence (CAPS) markers derived from nuc rDNA internal transcribed spacer (ITS1-5.8S-ITS2) and the mitochondrial intermediate peptidase (MIP) gene were developed to identify the homokaryons in *Agaricus sinodeliciosus* (Ling et al., 2019). It is necessary to develop a method for identifying the homokaryons in *W. cocos.*

In the present study, fruiting bodies of *W. cocos* were induced. Nuclear fluorescence staining was performed to determine the ratio of uninucleate and binucleate basidiospores in *W. cocos*. Based on the growth, fruiting characteristics, and mating reaction of different SSIs, the putative homokaryons and related characteristics were confirmed. Molecular markers derived from the SSR primer screening could distinguish homokaryons and heterokaryons, and genome heterozygous ratio analysis confirmed the results. The predominant mating system of *W. cocos* in the Chinese population was pseudo-homothallism; however, homokaryons in SSIs suggested that there was also a heterothallism mating system. Lastly, we proposed different breeding strategies based on the discovery of homokaryons that will be helpful for the screening of excellent cultivars and industrial development of *W. cocos*.

## Materials and Methods

### Fungal Materials

Ten *W. cocos* strains were used in this study. Strains 5.78 and Z1 were kindly provided by Hubei Provincial Hospital of Traditional Chinese Medicine. Strains CGMCC 5.137, 5.157, 5.908, 5.55, 5.545, and 5.2216 were collected from the China General Microbiological Culture Collection Center (CGMCC). Strain NBRC 30628 was collected from the Biological Resource Center (NBRC, NITE), and strain 28-1 was collected from Xixiang Edible Mushroom Institute, Shanxi province, China. The information of all the strains used is listed in Supplementary Table S1. All strains were maintained on potato dextrose agar medium (PDA: 200 g/L potato, 20 g/L glucose, 18 g/L agar) at 4°C. The identity of the strains was confirmed by DNA sequence analysis, through which the internal transcribed spacer (ITS) of nuclear ribosomal DNA was amplified and sequenced. ITS sequences of all the tested strains were deposited in GenBank under the accession numbers listed in Supplementary Table S1.

### Fruiting Bodies Induction

All strains were cultured on PDA plates for 5 days and then transferred to glass flasks (250 mL) containing 100 mL of improved PDA medium (PDA supplemented with KH_2_PO_4_ 1 g/L, MgSO_4_.7H_2_O 0.5 g/L, and VB_1_ 10 mg/L; Zeng et al., 2018). The cultures were placed at 25°C for 6 days under dark conditions and then irradiated continuously at an intensity of 400–500 lx for fruiting. The basidia and spores were observed using a scanning electron microscope (Hitachi SU8010, Tokyo, Japan), following the method of Khaund and Joshi (2014).

### DAPI Staining of Basidiospores

The strains CGMCC 5.2216, CGMCC 5.545, and Z1 were selected randomly for DAPI staining of basidiospores. The fruiting bodies were removed from the medium and transferred to a 5 mL centrifuge tube with 3 mL sterile water. The spore suspension was harvested by stirring fruiting bodies with forceps in the tube and then filtered using sterile absorbent cotton. For 4, 6-diamidino-2-phenylindole (DAPI) staining, 3–4 μL spore suspension was mixed with 1 μL of 5 μg/mL DAPI solution (Gen-View, El Monte, CA, United States), followed by incubation for 1 min in dark. The samples were observed under a fluorescence microscope (Axioimager A2, Zeiss, Göttingen, Germany) with an excitation wavelength of 340 nm and an emission wavelength of 488 nm. The number of nuclei of each basidiospore was observed and the ratios of basidiospores with different numbers of nuclei were counted.

### Isolation of Single Spore Isolates

The basidiospore suspension obtained as described above was diluted to 1 × 10^3^ spores/mL using sterile water, evenly spread 150 μL on the PDA Petri dishes (90 mm), and then incubated at 25°C for 4–10 days until spores germinated. SSIs were taken out using sterilized toothpicks when the germinated mono-colony was visible and subcultured individually on PDA plates at 25°C. SSIs from the parent strain CGMCC 5.545 were obtained.

### Study of Culture Characteristics of SSIs

Colony morphology, growth rates on PDA plates, and sawdust media of parent strain and SSIs were observed and compared. The Petri dishes were inoculated with agar plugs (8 mm diameter) at the center and cultured at 25°C. The method of cross line was applied according to the inoculation spot (Hanna, 1985) to record the position of the colony edge every 24 h for 4 days. The growth rate of each isolate at the PDA plate was calculated by dividing the average colony diameter by the growth days. Colony morphology was observed 15 days after inoculation. The pH of the PDA medium was measured 2 months after inoculation. The PDA media were cut into 4 × 4 mm blocks and transferred to 1.5 mL tubes. The tubes were placed in a water bath at 100°C until the media melted. The pH value was measured using pH indicator strips. For each strain, five plates were used to calculate the growth rates and measure the pH.

The growth rates in sawdust medium were studied in 20 × 200 mm tubes with evenly mixed pine sawdust substrate with 50% water content. The substrate comprised 78% pine sawdust (*Pinus sylvestris*, mixed with sapwood and heartwood), 20% wheat bran, 1% sucrose, and 1% gypsum. Spawn blocks 10 mm in diameter were inoculated on the surface of the substrate and compacted horizontally. All tubes were kept vertically and incubated at 25°C. The position of the colony edge was recorded every 48 h for 16 days after the spawn block germinated. The growth rate was calculated as the distance from the inoculated spot divided by the number of growth days (Siwul et al., 2009). For each strain, five tubes were used.

### Pairing Experiments

A one-to-one pairing and co-culture of several strains in a plate were conducted. PDA plates (90 × 90 mm) were inoculated with agar plugs (8 mm diameter) at 1.5 cm from the center (Punja and Grogan, 1983). All plates were incubated at 25°C until the colonies of different SSIs were in contact with each other. All the pairing experiments were repeated thrice.

### Distinguishing Homokaryons and Heterokaryons by Molecular Markers

Based on the genome of strain 5.78 (accession number of NCBI: PRJNA644235), the SSRs were searched using MISA^2^ (Beier et al., 2017) in view of perl scripts (Wei et al., 2015). SSR loci were randomly selected from the whole genome, and SSR loci with long repeat cores (three nucleotides or more) and more repeats were preferred. The selected SSRs were used to design primers using Oligo 7 software (Rychlik, 2007) according to their flanking regions. A total of 40 primer pairs were designed with a range of 18–24 bp to yield 100–350 bp amplification products. Primers were synthesized by Sangon Biotech Co. Ltd. (Shanghai, China) and the sequences are listed in Supplementary Table S2.

Mycelia for DNA extraction were harvested on PDA medium with cellophane after being cultured at 25°C for 15 days. Total genomic DNA was extracted following a modified cetyltrimethylammonium bromide method (Doyle and Doyle, 1987). The quality of the genomic DNA was determined by electrophoresis on a 1.0% agarose gel, and the concentration was determined using NanoDrop Lite Microvolume Spectrophotometer (Thermo Fisher Scientific, Waltham, MA, United States). The DNA solution was stored at -20°C for further use.

The PCR reactions were performed with a volume of 20 μL, containing 1 μL DNA (10 ng), 0.5 μL forward and reverse primers (10 μmol/L), 10 μL 2× mix (Vazyme, Nanjing, China), and 8 μL ddH_2_O. The PCR reaction conditions were as follows: 95°C for 3 min, followed by 35 cycles of 30 s at 95°C, 30 s at 48–56°C (annealing temperature depends on the primer pairs, which is listed in Supplementary Table S2), 30 s at 72°C, then extended at 72°C for 10 min and stored at 4°C. The amplified products were separated by polyacrylamide gel electrophoresis (PAGE) and photoed in the Tanon 1600 Gel Imager (Shanghai, China). All the PAGE experiments were repeated thrice.

### Illumina Sequencing of Tested Isolates

The putative homokaryotic (CGMCC 5.545 SS20), heterokaryotic strains (5.78 SS40, 5.78 SS46, and 5.78 SS84), and parent strain 5.78 were fermented in potato broth for DNA extraction. The mycelia were transferred to 50 mL sterile centrifuge tubes, washed twice with sterile water, and stored at -80°C until DNA extraction. Total DNA of *W. cocos* strain was extracted using blood and cell culture DNA midi kit (Q13343, QIAGEN, Dusseldorf, Germany). DNA degradation and contamination were monitored on 1% agarose gels. DNA purity was checked using the NanoPhotometer^®^spectrophotometer (IMPLEN, CA, United States) and concentration was measured using Qubit^®^DNA Assay Kit in Qubit^®^2.0 Flurometer (Life Technologies, CA, United States). The qualified samples were sequenced using the Illumina NovaSeq platform in Nextomics Biosciences (Wuhan, China). Clean reads were obtained by eliminating unmatched reads and low-quality reads, connector contamination, and duplication reads by *fastp*^3^ (Chen et al., 2018). Quality control was performed using FastQC^4^. Clean data (accession number of NCBI: PRJNA644235) were used to estimate the heterozygous ratio based on analysis of k-mer depth distribution using Jellyfish^5^ (Marcais and Kingsford, 2011) and GenomeScope^6^ (Liu et al., 2013; Vurture et al., 2017).

### Statistical and Correlation Analyses

Growth rates were expressed as the mean ± standard deviation (SD). Tests of significant differences were determined by Duncan’s multiple range test at *p* = 0.05 by one-way analysis of variance of the data (ANOVA) using SPSS Statistics 23 (International Business Machines Corporation, New York, NY, United States). Figures were generated using GraphPad Prism v8.0 (GraphPad Software Inc., San Diego, CA, United States).

Correlation analyses between different characteristics and karyotypes were performed using factor analysis using SPSS Statistics 23. First, each characteristic was assigned with different values, which are listed in Supplementary Table S4. Correlation coefficients were calculated, and a correlation matrix was drawn.

## Results

### Fruiting Body Induction

**FIGURE 1:**
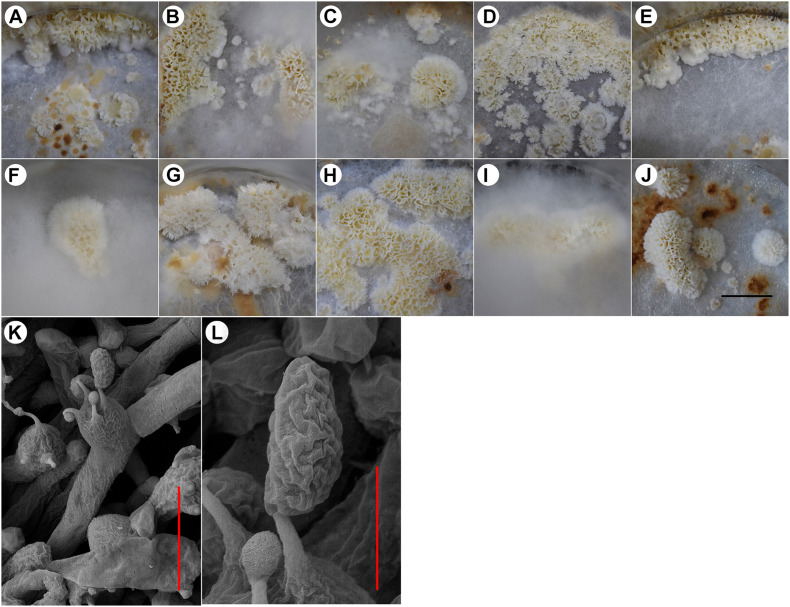
The fruiting bodies of different *W. cocos* strains. **(A)** strain 5.78, **(B)** strain Z1, **(C)** strain CGMCC 5.137, **(D)** strain CGMCC 5.157, **(E)** strain CGMCC 5.908, **(F)** strain CGMCC 5.55, **(G)** strain NBRC 30628, **(H)** strain CGMCC 5.545, **(I)** strain 28-1, **(J)** strain CGMCC 5.2216, **(K)** basidia of strain CGMCC 5.545, and **(L)** basidiospores of strain CGMCC 5.545. Scale bars: A–J = 2 cm, K: 10 μm, and L: 3 μm.

All 10 tested strains formed fruiting bodies under the designed culture conditions (Figure 1). The fruiting bodies of most strains were whitish resupinate poroids; however, there were some differences. Some strains formed fruiting bodies under aerial mycelia (Figures 1F,I) and some around the inner wall of the flask (Figures 1A,E). The strain NBRC 30628 and CGMCC 5.545 began to form fruiting bodies at 22–25 days after inoculation, but the majority of strains began to form fruiting bodies on approximately day 35.

The basidia and spores were observed in the fruiting bodies of all the above strains. Basidia clavata, the majority with four sterigmata (Figure 1K), basidiospores cylindrical to oblong-ellipsoid, thin-walled, 6.40–8.59 × 3.37–4.83 μm, with an average length of 7.39 μm, width of 3.87 μm, and ratio of length to width of 1.65–2.17 (Figure 1L).

### The Number of Nuclei in Basidiospores of *W. cocos*

**FIGURE 2:**
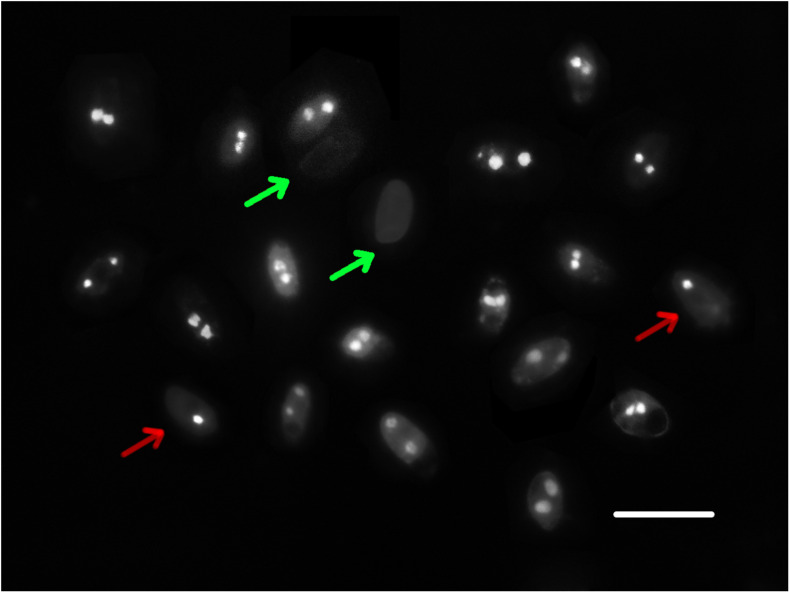
Nuclei of basidiospores of strain CGMCC 5.545. The red arrow indicates uninucleate spores, the green arrow indicates nuclear-free spores, and the others are binucleate spores. Scale bar: 10 μm.

A total of 123 basidiospores of strain CGMCC 5.2216 stained by DAPI were observed under a fluorescence microscope. Basidiospores with uni- or binucleate and nuclear-free were observed (Figure 2), with ratios of 20.3%, 67.5%, and 12.2%, respectively. In the other strains, Z1 and CGMCC 5.545, 55, and 36 basidiospores were observed, respectively, and the uninucleate, binucleate, and nuclear-free basidiospores were found with ratios of 25.5%, 50.9%, 23.6% and 9.7%, 80.6%, and 9.7%, respectively. All the tested strains showed that binucleate spores occupied the majority.

### Comparison of Colony Morphology and Growth Rates of SSIs in PDA Media

**FIGURE 3:**
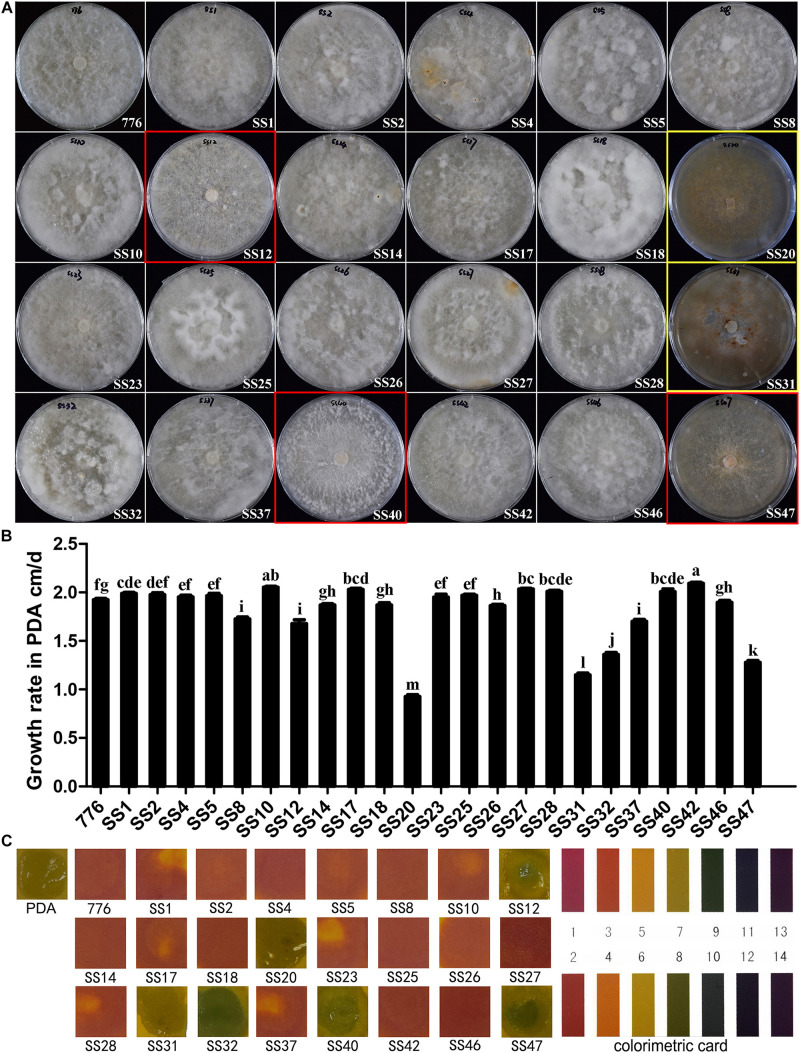
Colony morphology and growth of SSIs in PDA media. **(A)** Colony of different SSIs and parent strains cultured in PDA medium. White frame: parent-type strain, red frame: creeping-type strain, and yellow frame: brown-type strain. **(B)** Growth rates of different SSIs and parent strain. Different letters above the bars indicate the significant difference at *p* = 0.05 by Duncan’s multiple range tests. **(C)** pH of PDA media in which different SSIs and parent strain grew for 60 days. The pH was measured using pH indicator strips.

The 23 SSIs derived from the parent strain CGMCC 5.545 were involved in the comparison of colony morphology and growth rate. The majority of the SSIs (18/23) had white and dense aerial mycelia, which were the same as the parent strain (indicated with a white frame in Figure 3A). Some strains had fewer aerial mycelia creeping on the media (red frame in Figure 3A). Other strains had obvious brown mycelia, and the whole colony was brown with a fish-like smell (yellow frame in Figure 3A). Therefore, the SSIs were classified into three groups: parent-type, creeping-type, and brown-type.

There was a significant difference in the growth rates among the SSIs. About seven SSIs grew significantly faster than the parent strain (*p* < 0.05), and 8 SSIs showed a similar growth rate as the parent strain. The growth of the other eight SSIs was significantly slower than that of the parent strain CGMCC 5.545 (*p* < 0.05). The growth rates of strains SS20 and SS31 were even lower than that of half of the parent strain.

pH values of the PDA media in which the different SSIs were cultured for 60 days were determined by pH indicator strips, and the colors are shown in Figure 3C. The pH value of the medium of parent strain CGMCC 5.545 fell to 2–3, and the majority of strains (17/23) showed the same color as the parent strain. However, the pH values of the media increased to 8–9 after the growth of some SSIs of *W. cocos* (SS12, SS32, SS40, SS47; Figure 3C), and the pH values of the media of SS20 and SS31 was neutral (Figure 3C), which was the same as that of PDA media without inoculation.

### Comparison of the Growth of SSIs in Sawdust Substrate

**FIGURE 4:**
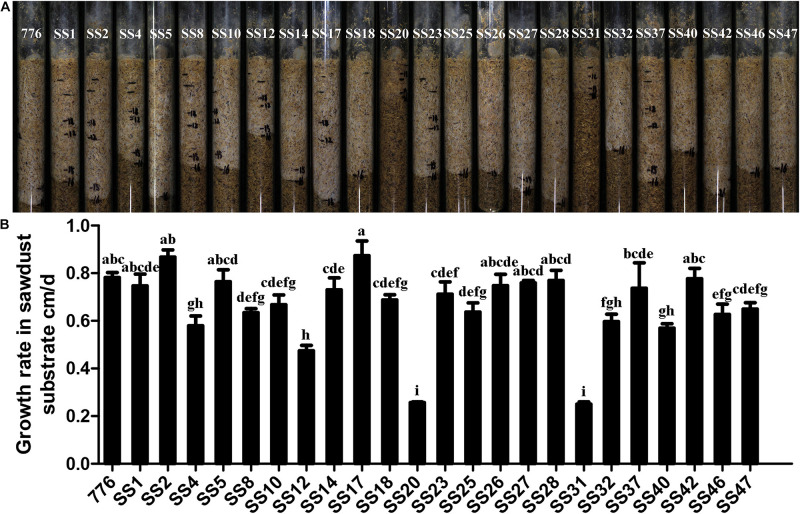
Growth in sawdust substrate of different SSIs and parent strains. **(A)** Growth condition of the sawdust substrate. **(B)** Growth rates in the sawdust substrate. Values with different letters above the bars were significantly different (*p* < 0.05) by Duncan’s multiple range tests.

The growth of 23 SSIs and the parent strain CGMCC 5.545 in the sawdust substrate is shown in Figure 4. The growth rates of the majority of strains (14/23) showed no difference with the parent strain, whereas the other strains grew significantly slower than that of the parent strain CGMCC 5.545. Strain SS20 and SS31 grew most slowly, and the growth rates were only one-third of the parent strain CGMCC 5.545. About 21 SSIs exhibited white and dense mycelia in the pine sawdust substrate, which was the same as the parent strain, but the mycelia of SS20 and SS31 were weak, indicating poor growth performance in the pine sawdust substrate.

### Fruiting of SSIs

Fruiting tests of 23 SSIs and the parent strain CGMCC 5.545 were performed in tubes or flasks. About five SSIs (SS12, SS20, SS31, SS40, and SS47; 21.7%) did not fruit even when the culture lasted for 120 days, and the others fruited in 57–90 days. The basidiospores of all fruiting bodies were observed under a microscope, and there was no difference with that of the parent strain. It was found that all the strains of creeping-type and brown-type cannot fruit, but all the parent-type strains formed fruiting bodies.

**FIGURE 5:**
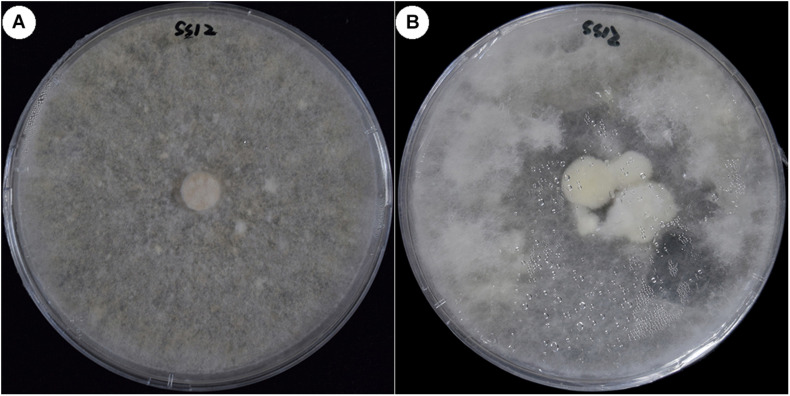
Fruiting of strain SS12 cultured in the medium with fermentation broth. **(A)** Colony of non-fruiting strain SS12 cultured in PDA medium. **(B)** SS12 fruited cultured in the PDA medium with fermentation broth of CGMCC 5.454.

For the five non-fruiting SSIs, many methods were used to induce the fruiting body. When the fermentation broth of the parent strain CGMCC 5.545 was added to the culture media, strain SS12 fruited (Figure 5), but others did not.

**FIGURE 6:**
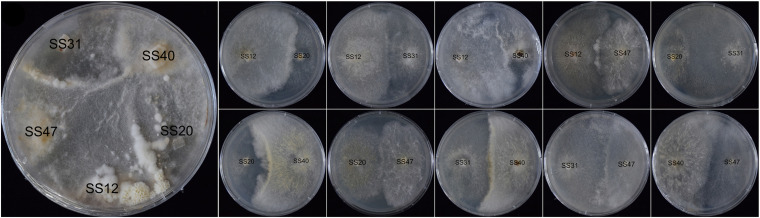
Mating reactions of non-fruiting SSIs.

The five non-fruiting SSI cultures under the tested conditions crossed each other in all possible combinations for a total of 10 crosses. No fruiting bodies were formed by pairwise coupling (Figure 6). However, when the five SSIs were co-cultured in a plate, fruiting bodies formed at the contact zone between SS31 and SS47, SS12, and SS20.

### Distinguishing Homokaryons and Heterokaryons by Molecular Markers

A total of 1612 SSR loci (one to six nucleotides) were identified in the genome of strain 5.78 (Supplementary Table S3). Trinucleotide SSRs were the most abundant microsatellite (40.7%), followed by dinucleotide (37.3%), mononucleotide (16.1%), hexanucleotide (3.0%), tetranucleotide (1.7%), and pentanucleotide (1.2%) (Supplementary Figure S2A). The most abundant SSR loci were dinucleotide AG/CT with a total of 325 (20% of the total SSRs), followed by C/G, CG/CG, and CCG/CGG. Only seven motif types had a number greater than 100 (Supplementary Figure S2B).

A total of 40 primer pairs were synthesized and named in sequential order of design. In total, five SSIs, including different-type strains according to their colony morphologies, were selected to conduct the primary screening. The products of primer pair SSR37, 38 exhibited various degrees of polymorphism among the five SSIs (Supplementary Figure S3A) and were considered putative markers for distinguishing homokaryons and heterokaryons.

The marker was further verified in the parent strain and all the SSIs. The results of PAGE exhibited that SS20 and SS31 had the same bands, being different from other SSIs and the parent strain when the selected marker was used (Supplementary Figure S3B). Combining the culture characteristics, SS20 and SS31 were the putative homokaryons for the next verification.

The primer SSR37, 38 was also used for testing the other nine parent strains whose fruiting bodies were induced in this study. The majority of the tested strains showed the same bands as the strain CGMCC 5.545 (Supplementary Figure S4A). The marker was applied to the 20 other putative homokaryotic strains screened based on culture characteristics. All the putative homokaryotic strains showed the similar band type with SS20 and SS31 (Supplementary Figure S4B).

### Heterozygosity of Putative Homokaryotic and Heterokaryotic Isolates Revealed by Illumina Sequencing

**FIGURE 7:**
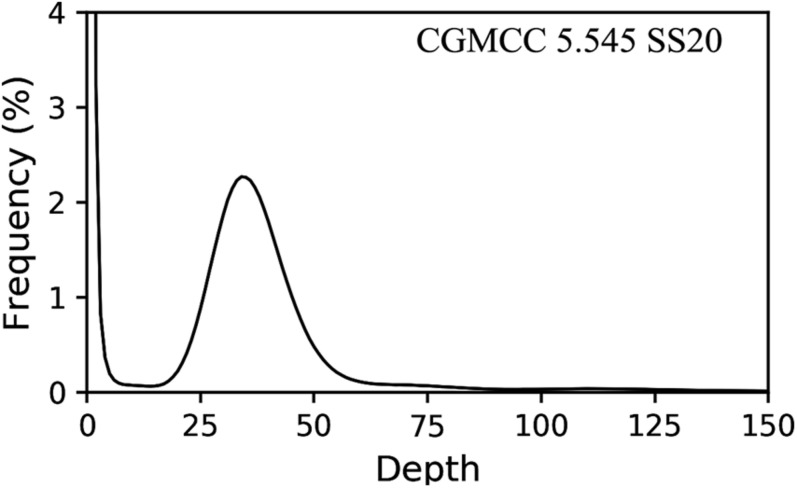
Heterozygous ratio of homokaryon CGMCC 5.545 SS20.

The putative homokaryotic strain CGMCC 5.545 SS20, heterokaryotic strain 5.78 SS40, 5.78 SS46, 5.78 SS84, and parent strain 5.78 were sequenced by the Illumina platform for evaluation of the heterozygous ratio. The results of k-mer analysis revealed obvious double peaks in the parent strain and heterokaryotic strains and a single peak in the putative homokaryotic strain (Figure 7). Heterozygous ratio of heterokaryotic strains 5.78, 5.78 SS40, 5.78 SS46, and 5.78 SS84 (Supplementary Figure S5) was approximately 0.75% and that of the homokaryotic strain SS20 was 0.01%.

### Correlation Analysis of Different Characteristics and Karyotypes

Correlation analysis was conducted among different culture characteristics and karyotypes using the 23 SSIs of strain CGMCC 5.545. The results revealed that colony type (0.843) had a significant positive correlation with karyotypes, followed by the growth rate in sawdust substrate (0.800) and PDA medium (0.757). It was indicated that colony type, growth rates in sawdust, and PDA medium were effective for identifying homokaryons. Among the different characteristics, colony type had a significant positive correlation with the growth rate in sawdust substrate (0.855) and fruiting capacities (0.931) (> 0.85). Media pH had a negative correlation with other characteristics. There was a significant positive correlation between growth rates in PDA medium and sawdust substrate. The growth rate in sawdust substrate was significantly and positively correlated with fruiting capacity (Table 1).

**TABLE 1:** Correlation analysis among different characteristics and karyotypes.

	**Colony type**	GR (PDA)*	GR (sawdust)**	**Media pH**	**Fruiting**	**Karyotype**
Colony type	1.000	0.783	0.855	−0.765	0.931	0.843
GR (PDA)*		1.000	0.771	−0.706	0.662	0.757
GR (sawdust)**			1.000	−0.679	0.742	0.800
Media pH				1.000	−0.866	−0.420
Fruiting					1.000	0.588
Karyotype						1.000

*Growth rate in PDA medium. **Growth rate in sawdust medium.

## Discussion

Homokaryon is the basic material for cross-breeding, and the existence of homokaryon and heterokaryon makes it difficult to determine the mating system of *W. cocos*. This report identified the homokaryon from numerous SSIs by comparing culture characteristics, fruiting tests, and molecular markers, which was further demonstrated by k-mer analysis based on Illumina sequencing of strain SS20. Brown-colony type, slow growth rate, and neutral pH of the medium after being cultured were found to be the morphological and growth characteristics to distinguish homo- and heterokaryotic strains. Fruiting, pairing tests, and molecular markers provided more evidence, and homozygosity analysis of putative homokaryotic strains confirmed our results. This is the first report of distinguishing homokaryotic and heterokaryotic isolates combining cultures with genetic characteristics in *W. cocos*, and it will be helpful for crossbreeding of this precious medicinal mushroom and understanding the evolution and population structure.

Unlike most basidiomycetes, sclerotia are important edible and medicinal parts for *W. cocos*. The fruiting body of *W. cocos* is rarely found both in nature and sclerotium cultivation; however, the fruiting body is necessary for genetic research and cross-breeding. In the early stage, Tabata and Hiraota (1994) discovered that some mycelia and sclerotia of *W. cocos* can form fruiting bodies. Xu et al. (2014) reported that temperature, light, and wounding had obvious effects on fruiting. Korean strains form fruiting bodies in Petri dishes under suitable conditions (Jo et al., 2017). In the present study, all the strains tested could form fruiting bodies under our optimized conditions, which was the foundation of the fruiting trial in SSIs.

Basidiospores of *W. cocos* with uninucleate, binucleate, and nuclear-free were observed, which is consistent with the results of Xiong et al. (2006) and Li et al. (2002). Different ratios were observed in the three tested strains, but the same trend was observed for the binucleate basidiospores. Except for *A. bisporus*, basidiospores from fungi in *Amanita*, Cortinariaceae, and *Laccaria* were primarily binucleate (Horton, 2006). The binucleate spores can germinate to form self-fertile, heterokaryotic mycelia that can complete the sexual cycle in solo culture (pseudohomothallic inbreeding) or segregate hyphae of opposite mating type to enable outcrossing (Ni et al., 2011). Thus, distinguishing homokaryons and heterokaryons is important for *W. cocos*.

In this study, homokaryons were identified by combining culture characteristics and molecular markers. Among the 23 SSIs derived from the parent strain CGMCC 5.545, strains SS20 and SS31 grew weakly and most slowly as brown-type and showed poor performance on both PDA media and sawdust substrates. Fruiting trials revealed that they could not form fruiting bodies under the tested conditions. These were putative homokaryons. Moreover, SS20 and SS31 had the same bands, which were different from other SSIs and the parent strain when primer pair SSR 37, 38 was used. Illumina sequencing demonstrated that the heterozygous ratio of the putative homokaryotic strain SS20 was 0.01%, whereas the heterokaryotic strain was 0.75%. Based on the above evidence, strains SS20 and SS31 should be homokaryons. It was proposed that brown-type mycelia (brown mycelia with a special odor) and slow growth in both PDA media and sawdust substrates could be used as the primary judgment for homokaryons in *W. cocos*. The frequency of putative homokaryotic SSIs was about 8.3%, which was coincident with the frequency of uninucleate basidiospores (9.7%) in the strain CGMCC 5.454. It seemed that the homokaryotic SSIs should be derived from uninucleate basidiospores.

Non-fruiting strain SS12 fruited when the fermentation broth of the parent strain CGMCC 5.545 was added to the culture media (Figure 5), indicating that some special compounds or pH might be necessary for the fruiting of *W. cocos.* It was also suggested that not all the non-fruiting SSIs were homokaryons, which was different from that of *A. bisporus* (Sharma et al., 2014). Fruiting bodies were observed when the five non-fruiting SSIs were co-cultured in one plate, but no fruiting bodies formed when they were crossed in pairs (Figure 6). This may be because some compounds produced in the co-culture could promote the formation of fruiting bodies for some SSIs. Another potential scenario could be parasexual recombination between the co-existing strains, leading to the formation of a viable heterokaryon. Based on the culture characteristics and molecular marker analysis, strains SS12, SS40, and SS47 should be heterokaryons although they could not fruit under normal conditions.

Fruiting of SSIs is the most decisive experiment for the determination of mating system. In *W. cocos*, the fruiting ratio of SSIs was 78.3% in the strain CGMCC 5.545. Combining the ratio of heterokaryons, it was indicated that pseudo-homothallism was predominant in the Chinese population of *W. cocos*. James et al. (2013) reported that *W. cocos* was likely to be heterothallic with a bipolar mating system based on the greater polymorphism at HD locus than P/R locus. However, the strain used was from America, which has been reported as not conspecific with *W. cocos* from East Asia (Wu et al., 2020) and there were not any fruiting experiments. Xiang et al. (2016) reported that hybrids between SSIs of different Chinese *W. cocos* strains could fruit; however, they didn’t distinguish the homokaryon and heterokaryon. In our opinion, heterothallism should exist in *W. cocos*, but more proofs need to be provided.

**FIGURE 8:**
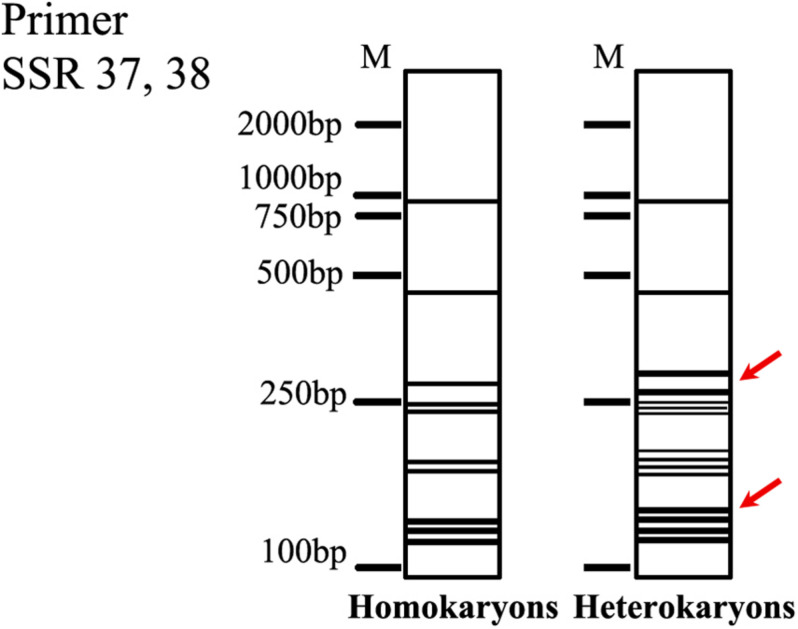
The fingerprints of the homokaryon of *W. cocos.* The red arrow indicated the key bands.

Genome-wide SSR mining has been successfully applied to *A. bisporus* (Foulongne-Oriol et al., 2009; Wang et al., 2019), *Ganoderma lucidum* (Qian et al., 2013), *V. volvacea* (Wang et al., 2014), and *Flammulina velutipes* (Liu et al., 2016) for genetic diversity, population structure, and strain identification. Generally, SSR analysis should generate single specific amplicons for homokaryons and two amplicons for heterokaryons. Molecular markers (SSR37, 38) derived from the SSR marker screening were confirmed to distinguish homokaryotic and heterokaryotic strains of *W. cocos* successfully in the present study although the results did not satisfy the rule of SSR. The fingerprints of the primer SSR 37, 38 were stable when more different homokaryotic and heterokaryotic strains were used (Supplementary Figure S4). According to the PCR results, we constructed a fingerprint to identify the homokaryon of *W. cocos* (Figure 8), which could serve as a reference for homokaryon identification of *W. cocos*. However, the fungal strains used in the present study originated from China, whereas the natural distribution range of this fungus is much broader. Furthermore, a wider screening of strains from different geographic regions would be needed to understand how widely applicable the marker is at the metapopulation level.

K-mer analysis, as the fundamental method, was always used to evaluate the heterozygous ratio and genome size in genome sequencing (Kajitani et al., 2014). The k-mer analysis was first used to verify homokaryotic and heterokaryotic strains, which provided a new method to further studies.

Based on the discovery of the homokaryon and putative mating system of *W. cocos*, we proposed a breeding strategy. First, we distinguished homokaryon and heterokaryon among SSIs derived from excellent parent strains by comparing cultivation characteristics and molecular marker analysis. Subsequently, heterokaryons of excellent parent strains can be used for sclerotium cultivation for screening, and homokaryons of different strains with excellent characteristics can be used to hybridize to combine the best traits. Heterokaryons are optional materials for the selection of excellent progenies that are also used in the breeding of *A. bisporus.* Crossbreeding based on homokaryons is an effective method that can integrate excellent characteristics from parent strains.

## Data Availability Statement

The datasets presented in this study can be found in online repositories. The names of the repository/repositories and accession number(s) can be found below: https://www.ncbi.nlm.nih.gov/, PRJNA644235.

## Author Contributions

SL and CD conceived and designed the experiments. SL performed the experiments and wrote the manuscript. QW and CD critically reviewed and curated the manuscript. QW and CD were responsible for the project. All authors contributed to the article and approved the submitted version.

## Conflict of Interest

The authors declare that the research was conducted in the absence of any commercial or financial relationships that could be construed as a potential conflict of interest.

## Footnotes

1 http://www.chyxx.com/shuju/202005/860533.html

2 https://webblast.ipk-gatersleben.de/misa/

3 https://github.com/OpenGene/fastp

4 http://www.bioinformatics.babraham.ac.uk/projects/fastqc

5 http://www.cbcb.umd.edu/software/jellyfish

6 https://github.com/schatzlab/genomescope.git.
