# Supplementary material for: Distinguishing Homokaryons and Heterokaryons in Medicinal Polypore Mushroom Wolfiporia cocos (Agaricomycetes) Based on Cultural and Genetic Characteristics
Source: Front Microbiol. 2021 Jan 25;11:596715. doi: 10.3389/fmicb.2020.596715 (PMC7868546; doi:10.3389/fmicb.2020.596715)
Supplement: Supplementary file 2 [file Data_Sheet_2.docx]

**Supplementary Figures**

Supplementary Figure 1


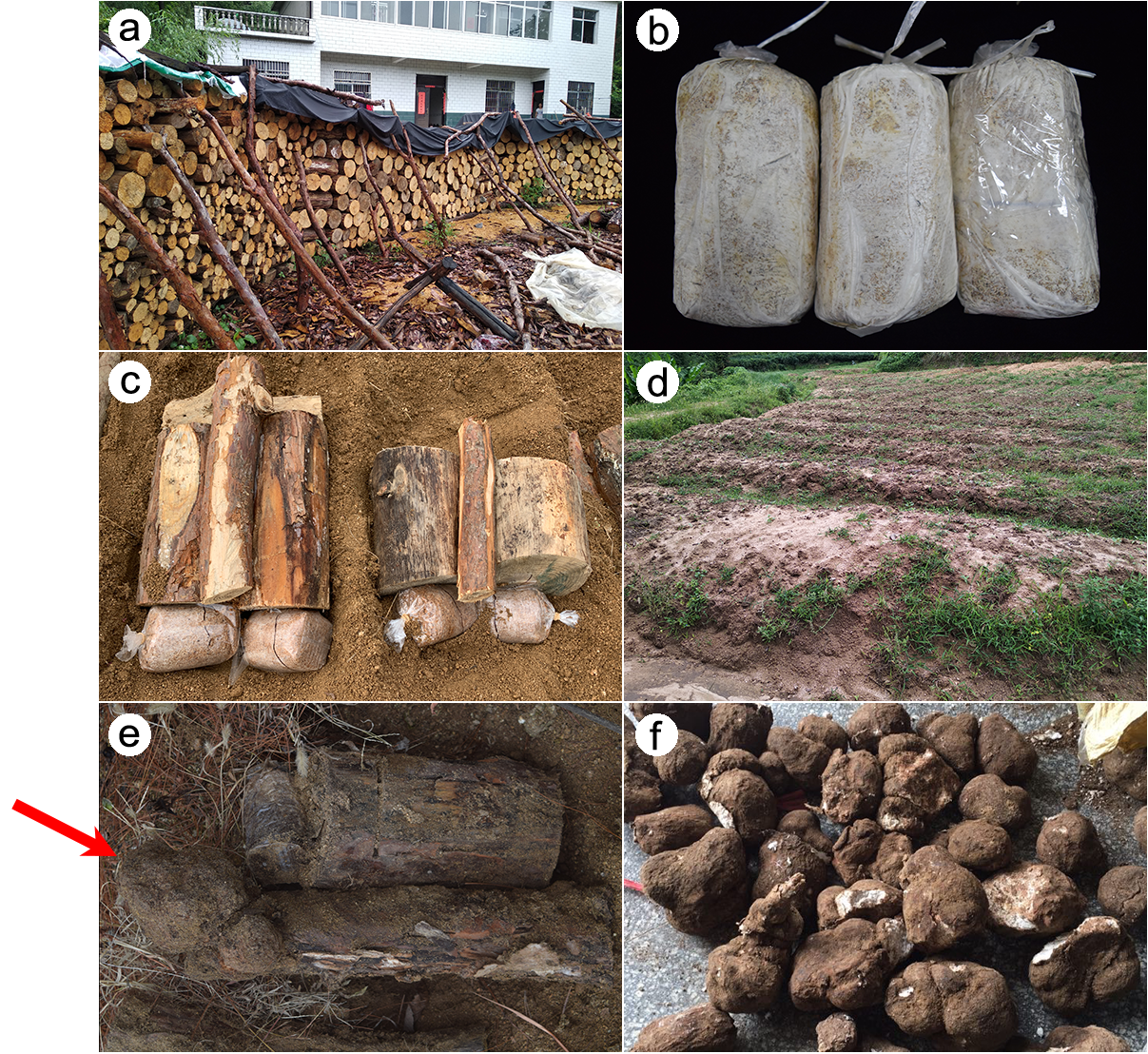


Fig. S1. Sclerotia cultivation of *W. cocos*

1. Pine log preparation; (b) Spawn preparation; (c) Inoculation; (d) Growth environment of *W. cocos* sclerotia; (e) Sclerotia harvesting; red arrow indicated the sclerotium of *W. cocos*; (f) Sclerotia

Supplementary Figure 2


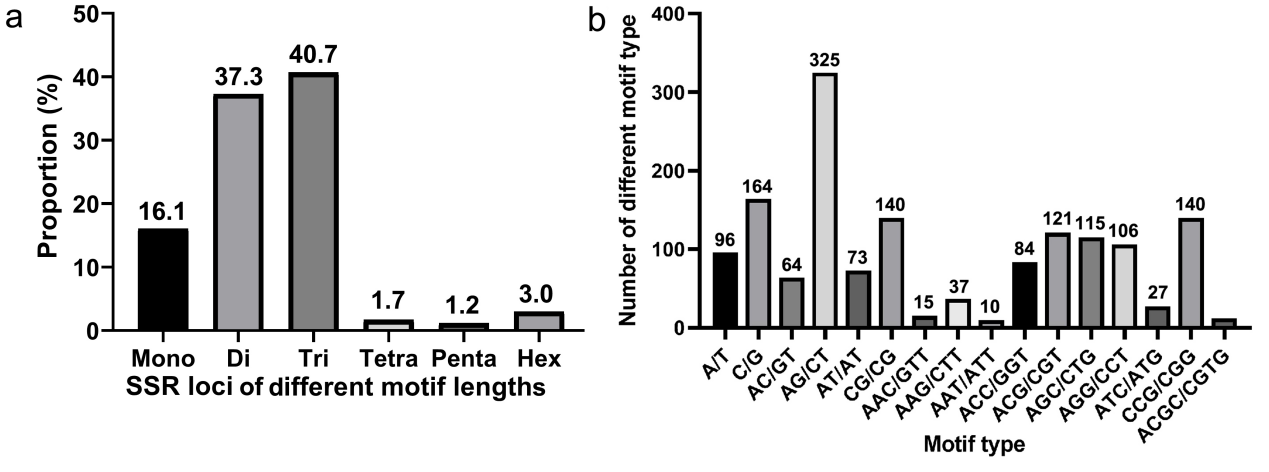


Fig. S2. Statistics of different motif lengths and motif types

(a) Proportion of SSR loci with different motif lengths; (b) The composition of different repeat motif in the genome of *W. cocos* (Number of motif type ≥10 were listed).

Supplementary Figure 3


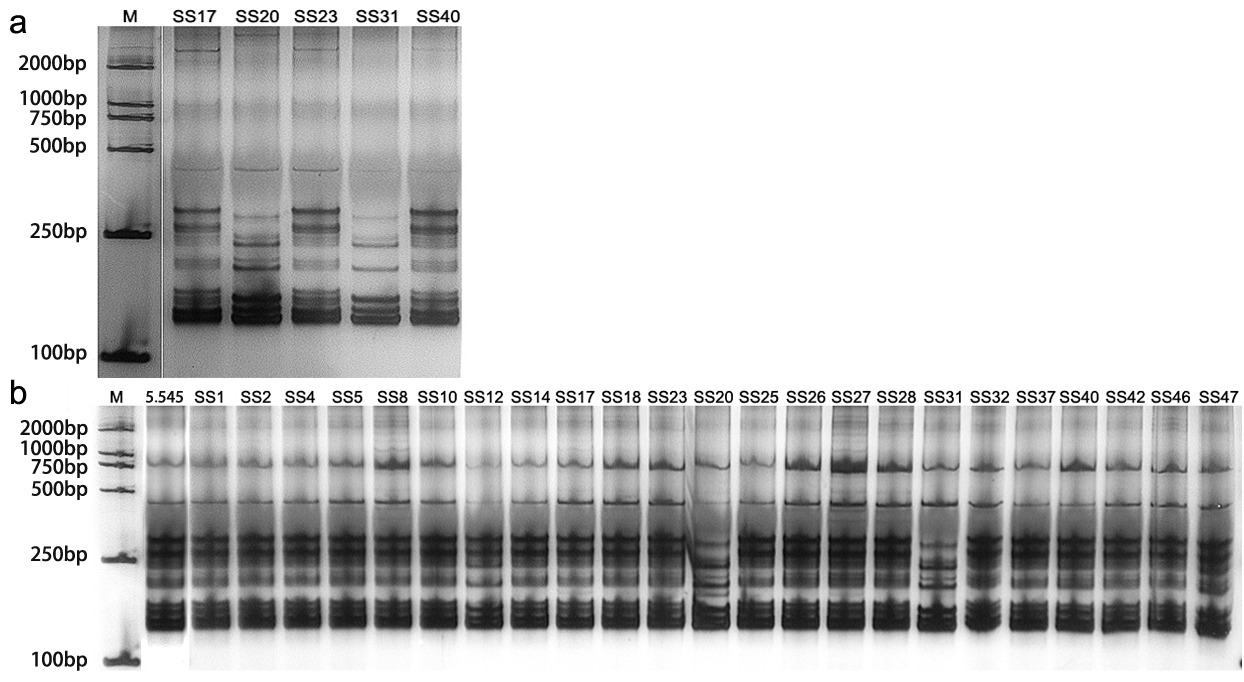


Fig. S3. Primer screening and distinguishing of homokaryotic and heterokaryotic SSIs of CGMCC 5.545.

(a) Screening of suitable primers.

SS17, 20, 23, 31 and 40 are SSIs derived from parent strain CGMCC 5.545. SS20 and 31 are the putative homokaryons according to the culture characteristics.

(b) Distinguishing of homokaryotic and heterokaryotic SSIs derived from parent strain CGMCC 5.545 by primer SSR37, 38.

SS20 and 31 are the putative homokaryons and others are heterokaryons.

Supplementary Figure 4


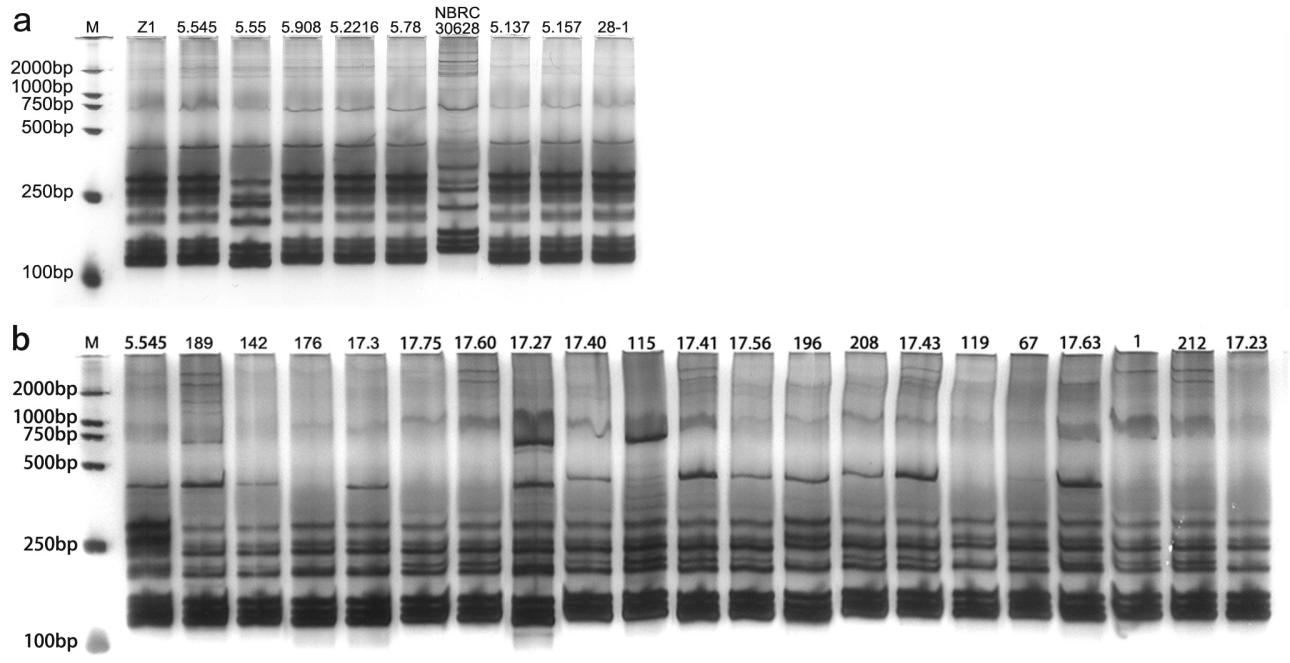


Fig. S4. Confirmation of the marker SSR 37, 38

(a) The marker SSR 37, 38 was used in the 10 parent strains of *W. cocos*.

All the strains exhibited the similar bands as heterokaryons and the information of the strains used are listed in Table S1.

(b) The marker SSR 37, 38 was used in different homokaryotic strains derived from parent strain CGMCC 5.545.

All the SSIs used are the putative homokaryotic strains which exhibited the similar bands as homokaryons.

Supplementary Figure 5


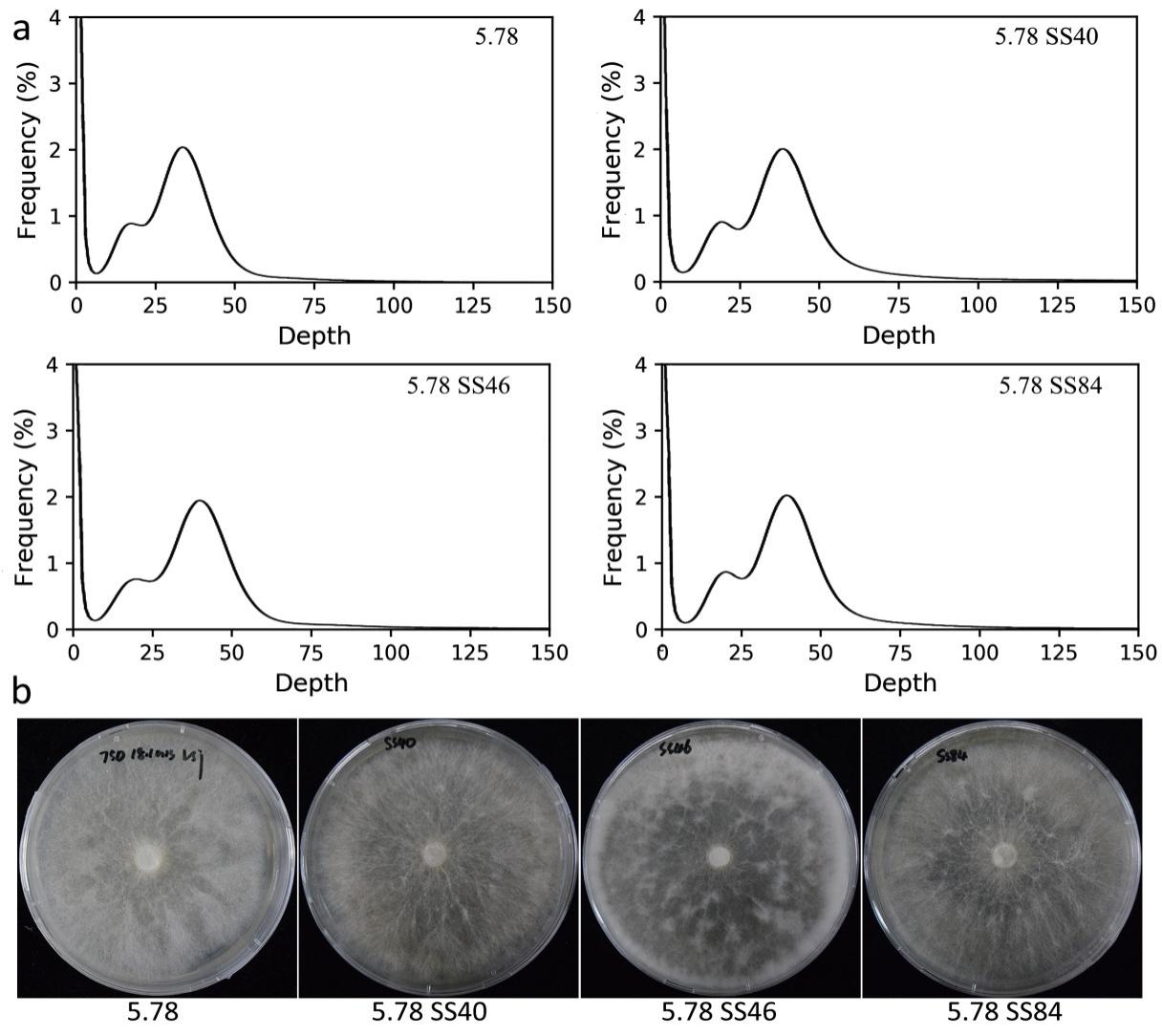


Fig. S5. Heterozygous ratio analysis of parent strain and heterokaryon SSIs. (a) Heterozygous ratio of parent strain 5.78 and heterokaryons 5.78 SS40, 5.78 SS46, 5.78 SS84. (b) Colony type of parent strain 5.78 and heterokaryons 5.78 SS40, 5.78 SS46, 5.78 SS84.
